# A Neuroimaging Audit of Patients Referred to an Old-Age Community Mental Health Team From Memory Assessment Service: Compliance With the Royal College of Radiologists Guidance

**DOI:** 10.7759/cureus.96682

**Published:** 2025-11-12

**Authors:** Hassan Manzoor

**Affiliations:** 1 Psychiatry, Birmingham and Solihull Mental Health NHS Foundation Trust, Birmingham, GBR

**Keywords:** alzheimer’s dementia, brain ct scan, dementia patients, psychiatry of old age, radiology report

## Abstract

Background

Neuroimaging is a crucial component in the assessment of patients with suspected dementia, aiding in diagnostic confirmation, subtype differentiation, and the exclusion of reversible causes. The Royal College of Radiologists (RCR) provides standards to guide the structured and comprehensive reporting of imaging scans, aiding in diagnostic accuracy and clinical decision-making. Local compliance with RCR standards may vary, which could influence the clinical utility of the scans. This audit was undertaken to evaluate local compliance with RCR reporting standards and identify areas for improvement.

Methodology

A total of 50 referrals from the Memory Assessment Service to the Old Age Community Mental Health Team (April 2024-September 2025) were reviewed. Radiology reports were analyzed for compliance with RCR standards, focusing on documentation of atrophy pattern, vascular pathology, reversible causes, use of rating scales, and inclusion of diagnostic conclusions. Data were also organized by imaging modality and indication to assess reporting consistency and completeness.

Results

Of the 50 referrals, nine (18%) patients were diagnosed with dementia without neuroimaging, consistent with clinical guidelines. Among 41 imaged patients, 33 had CT and four had MRI scans. Cerebral atrophy was described in 85% of reports, increasing to 100% for cognitive impairment cases; however, standardized scoring systems were infrequently used (Medial Temporal Atrophy score 7/22). Vascular pathology was mentioned in most reports with severity markers, but Fazekas scoring was used in 3/22 scans. Reversible causes were more comprehensively covered in scans done for indications other than cognitive impairment. Overall, 15/22 (68%) cognitive impairment reports included a diagnostic conclusion.

Conclusions

Local neuroimaging reports for dementia patients showed comprehensive recognition of atrophy and vascular changes but inconsistent use of standardized scoring, documentation of reversible causes, and diagnostic conclusions. Structured reporting templates and multidisciplinary feedback could improve adherence to RCR standards and enhance clinical utility.

## Introduction

Dementia is a progressive neurodegenerative condition that requires accurate diagnosis and subtyping to guide management and prognosis [1]. Neuroimaging plays a crucial role in this process by helping to identify potentially reversible causes, distinguish between dementia subtypes, and exclude other intracranial pathologies [2]; however, in specific situations, dementia can be diagnosed without neuroimaging, as highlighted by the Royal College of Psychiatrists guidelines [3].

The Royal College of Radiologists (RCR) has outlined reporting standards for the interpretation and reporting of imaging investigations [4]. Two standards that are pertinent to neuroimaging in dementia workup are: (1) An actionable imaging report should answer the clinical question asked by the referrer. (2) When an abnormality is described, a tentative or differential diagnosis should be provided.

Adherence to these standards ensures consistency, clarity, and diagnostic accuracy in reporting. In routine clinical practice, the completeness and quality of reporting can vary due to differing levels of experience, varying expectations, and underutilization of structured templates [5,6].

This audit was undertaken to assess local compliance of neuroimaging reports for patients who had been under the local specialist dementia service with the RCR reporting standards. The findings aim to identify gaps in practice and provide recommendations for improving the quality and standardization of imaging reports.

## Materials and methods

This audit was conducted at the Juniper Centre Old Age Community Mental Health Team (OACMHT) at Birmingham and Solihull Mental Health NHS Foundation Trust to assess the compliance of neuroimaging reports for dementia patients with the RCR reporting standards.

Study design and setting

A retrospective audit design was employed. A total of 50 patient referrals from the Specialist Memory Assessment Service (MAS) to the OACMHT between April 2024 and September 2025 were reviewed. All patients had been diagnosed with dementia by MAS and referred to OAMCHT for consideration of dementia medications.

Data sources and extraction

Radiology reports were accessed via the electronic medical record system RiO, using the Shared Care Records (SCR) interface. Reports were available in free-text format. Data were manually extracted and entered into a structured Microsoft Excel spreadsheet for analysis.

Audit parameters

Each report was examined for adherence to key elements recommended by the RCR standards for dementia-related neuroimaging reporting. The parameters that were assessed are mentioned in Table 1.

**Table 1:** Parameters analyzed for neuroimaging reports.

Parameters analyzed
Clinical indication for the scan
Imaging modality utilized (CT, MRI, or other)
Documentation of neurodegenerative features, including mention of generalized or lobar-specific atrophy, Medial Temporal Atrophy score, and Koedam score
Reference to vascular pathology, including description of small-vessel disease and Fazekas scoring, where applicable
Assessment for reversible causes of cognitive impairment, such as subdural haematoma, space-occupying lesions, or normal-pressure hydrocephalus
Whether the report provided an explicit or inferential answer to the clinical question posed by the referrer, including a tentative or differential diagnosis and/or summary conclusion

Data analysis

All extracted data were analyzed descriptively to determine the proportion of reports that met each audit criterion. Data extraction was performed by a single reviewer (the author), who manually reviewed all radiology reports. The results were compared against the RCR standards to evaluate overall compliance and identify areas for improvement.

## Results

Overall, nine out of 50 patients referred from MAS had been diagnosed with dementia without needing neuroimaging. This is in line with clinical guidelines where patients who are too advanced in their illness, or having severe functional decline, or having typical Alzheimer’s dementia (AD) symptoms without atypical features can be diagnosed without neuroimaging. For the rest of the 41 patients who had neuroimaging, either requested by MAS or those who had recent neuroimaging for other indications, their data were further analyzed.

Modality

Different modalities were utilized depending on the clinical need. Overall, 33 patients had CT scans, four had MRI scans, one each had MRA and CTA, and two had DAT scans.

Clinical indications

Neuroimaging was requested for both cognitive and non-cognitive indications in approximately equal proportions, with the non-cognitive group primarily including acute presentations such as falls and suspected stroke, among others. Table 2 breaks down the indications for scans.

**Table 2:** Breakdown of indications for the scans.

Indication	Percentage
Cognitive impairment	22/41 (53%)
Falls	8/41 (19.5%)
Stroke suspicion	4/41 (9.7%)
Lewy body dementia	2/42 (4.8%)
Mobility decline, metastasis, confusion, seizures, and arteriovenous malformation follow-up	1 each

Overall, 22/41 had neuroimaging for cognitive impairment. This was followed by 8/41 for falls, 4/41 for stroke suspicion, and 2/41 for query of Lewy body dementia. There was one scan each for the indication of mobility decline, metastasis, confusion, seizures, and arteriovenous malformation follow-up.

Radiological findings: cerebral atrophy and volume loss

Overall, 85% of scans referenced cerebral atrophy. This percentage increased to 100% for the 22 scans that were performed for the indication of cognitive impairment. For cognitive impairment scans, a pattern of atrophy was identified, as outlined in Table 3.

**Table 3:** Distribution of atrophy patterns.

Pattern of atrophy	Percentage
Medial temporal and hippocampal volume loss	7 (31%)
Generalized atrophy without predominance	5 (22%)
Temporal predominance	2 (9%)
Hippocampal predominance	1 (4%)

Use of Standardized Scoring

Standardized scoring systems were utilized sparingly. The Medial Temporal Atrophy (MTA) score was the most used score, followed by the Global Cortical Atrophy (GCA) score. Koedam scoring, which assesses the severity of parietal atrophy, was utilized in one scan. This is outlined in Table 4.

**Table 4:** Breakdown of utilization of cortical atrophy scores. MTA = Medial Temporal Atrophy; GCA = Global Cortical Atrophy

Scoring	Percentage	Notes
MTA score	7/22 scans	Scores: 0 (3), 2 (2), 3 (1), asymmetrical (1)
Koedam score	1/22 scans	score: 2
GCA score	5/22 scans	scores: 0, 1, 1, 2, 2

Radiological findings: vascular disease and severity

Scans for Cognitive Impairment

**Figure 1:**
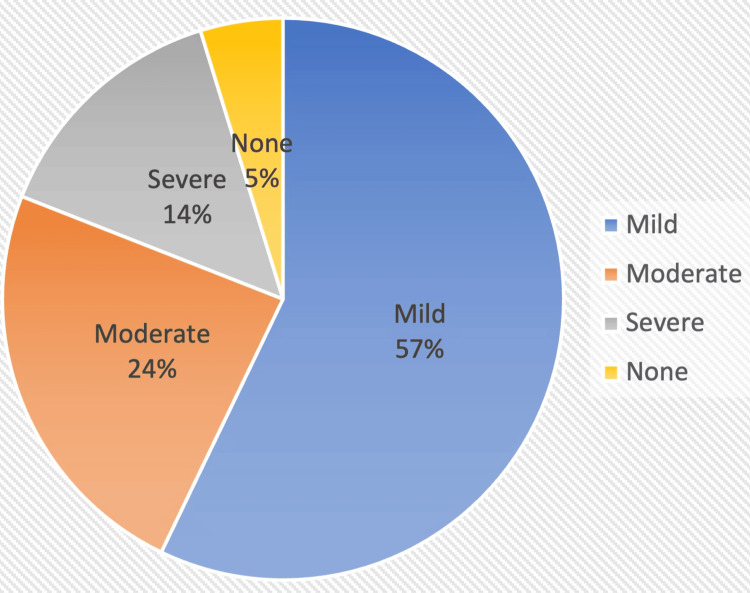
Vascular disease severity in scans for cognitive impairment.

Vascular changes were referenced in 21/22 of cognitive impairment scans. Severity distribution is depicted in Figure 1. Fazekas’ scoring was reported in 3/22 scans (scores: 0, 1, 2).

Scans for Other Indications

**Figure 2:**
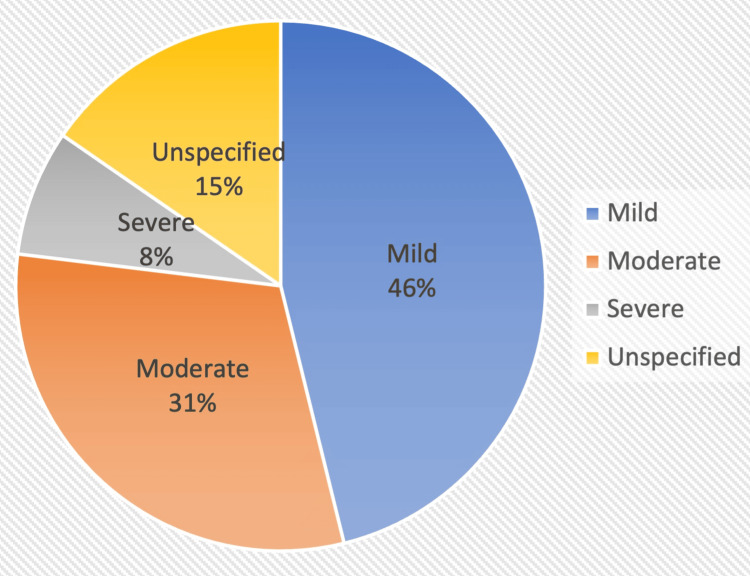
Vascular disease severity in scans for indications other than cognitive impairment.

For the rest of the scans, 13/19 referenced vascular disease. Figure 2 illustrates the severity distribution.

Exclusion of reversible causes

Reports were analyzed for explicit documentation regarding subdural hemorrhage/hematoma, space-occupying lesion, and normal-pressure hydrocephalus. For cognitive impairment scans, the coverage of reversible causes is summarized in Table 5.

**Table 5:** Coverage of reversible causes in scans for cognitive impairment.

Mention of reversible cause(s)	Coverage
Three	6/22 (27%)
Two	9/22 (41%)
One	1/22 (5%)
None	6/22 (27%)

The coverage of reversible causes was more comprehensive for other scans, where most had an acute indication. Table 6 depicts the coverage of reversible causes in these scans.

**Table 6:** Coverage of reversible causes in scans for indications other than cognitive impairment.

Mention of reversible cause(s)	Coverage
Three	8/19 (42%)
Two	6/19 (32%)
One	0
None	5/19 (26%)

Diagnostic conclusions in the reports

Scans for cognitive impairment had a conclusion rate of 68%. Overall, 2/22 (9%) reports did not provide any conclusion, and 5/22 (22%) restated the imaging findings. Of the 15 reports with conclusions, eight offered a specific diagnosis: AD (5), mixed AD/vascular dementia (2), Lewy body dementia, and Parkinson’s dementia (1 each). There were 19 scans done for other clinical indications, and all included a conclusion in their reports.

Incidental findings

A few scans had incidental findings. Arachnoid cysts were identified in three patients, and one patient had parafalcine encephalomalacia.

## Discussion

This audit demonstrates variable adherence of local neuroimaging reports for patients with suspected dementia to the RCR reporting standards. While most reports commented on global or regional atrophy and vascular changes, the documentation of standardized scoring systems and explicit diagnostic conclusions remained inconsistent.

Although 85% of reports referenced cerebral atrophy and 100% did so when the indication was cognitive impairment, a minority incorporated standardized rating scales such as the MTA or Koedam score. This is pertinent because structured scales would improve reproducibility and interobserver reliability. Their underutilization may reflect variable familiarity among radiologists with dementia-specific reporting frameworks or the absence of structured templates within the reporting system. Similarly, vascular pathology was widely described but not consistently graded using Fazekas scores.

Another gap was in the documentation of reversible causes such as subdural hematoma, space-occupying lesions, and normal-pressure hydrocephalus. Although all reports likely evaluated these implicitly, explicit mention provides reassurance to the referrer and supports comprehensive exclusion of secondary causes. Structured reporting templates that prompt mention of these elements could improve completeness without extending reporting time [7,8].

In line with RCR standards, an actionable report should both answer the referrer’s clinical question and provide a tentative or differential diagnosis when abnormalities are identified [4]. While most reports fulfilled the descriptive component, fewer than three-quarters included a clear diagnostic conclusion, and in several cases, findings were restated without interpretation, which can limit clinical utility, particularly in multidisciplinary memory clinics where radiological synthesis informs diagnosis and care planning.

The audit also highlights an appropriate degree of clinical judgment at the referral stage. Nine patients (18%) were diagnosed with dementia without neuroimaging, consistent with national guidance allowing clinical diagnosis in advanced or typical cases. This suggests good awareness among referring teams of when imaging meaningfully contributes to assessment.

This audit was limited by its retrospective single-site design and reliance on existing radiology reports, which may not fully reflect the quality of communication or undocumented clinical discussions. In many cases, imaging is reported remotely by radiologists from other organizations, making it difficult to implement a standardized reporting process. The study also did not assess inter-rater reliability or evaluate the clinical impact of reporting quality on patient management. These limitations may have influenced the findings by introducing observer bias in data extraction, restricting generalizability to other services or radiology departments, and relying on documentation that may not fully capture clinical communication. The retrospective nature also limited the ability to validate or clarify ambiguous report elements.

## Conclusions

Overall, this audit highlights opportunities to enhance neuroimaging report quality through increased use of validated scoring systems, systematic coverage of key pathologies, and consistent provision of diagnostic interpretation aligned with RCR standards. The results indicate that while local practice captures most core descriptive elements of dementia imaging, there is scope for improvement. Introducing a standardized dementia imaging reporting proforma could enhance both completeness and consistency. Regular multidisciplinary feedback sessions between radiologists and memory service clinicians would also help share the expectations that referring clinicians have of their radiologist colleagues. The findings will be shared with the local radiology team, and their feedback will be welcomed. A re-audit following implementation of such measures would determine their impact on the reporting.
